# Female and male mice have differential longterm cardiorenal outcomes following a matched degree of ischemia–reperfusion acute kidney injury

**DOI:** 10.1038/s41598-021-04701-x

**Published:** 2022-01-12

**Authors:** Danielle E. Soranno, Peter Baker, Lara Kirkbride-Romeo, Sara A. Wennersten, Kathy Ding, Brysen Keith, Maria A. Cavasin, Christopher Altmann, Rushita A. Bagchi, Korey R. Haefner, John Montford, Katja M. Gist, Laurent Vergnes, Karen Reue, Zhibin He, Hanan Elajaili, Kayo Okamura, Eva Nozik, Timothy A. McKinsey, Sarah Faubel

**Affiliations:** 1 Department of Pediatrics, Pediatric Nephrology, University of Colorado, 13123 E. 16th Ave, Box #328, Aurora, CO 80045 USA; 2 Division of Renal Disease and Hypertension, Department of Medicine, University of Colorado, Aurora, CO USA; 3 Consortium for Fibrosis Research & Translation, University of Colorado, Aurora, CO USA; 4 Department of Bioengineering, University of Colorado, Aurora, CO USA; 5 Department of Pediatrics, Clinical Genetics & Metabolism, University of Colorado, Aurora, CO USA; 6 Division of Cardiology, Department of Medicine, University of Colorado, Aurora, CO USA; 7 School of Medicine, University of Colorado, Aurora, CO USA; 8 Rocky Mountain Regional Veterans Affairs Medical Center, Aurora, CO USA; 9 Department of Pediatrics, Pediatric Cardiology, Cincinnati Children’s Hospital, University of Cincinnati, Cincinnati, OH USA; 10 Department of Human Genetics and Metabolism Theme Area, University of California Los Angeles, Los Angeles, CA USA; 11 Department of Pediatrics, Critical Care Medicine, University of Colorado, Aurora, CO USA

**Keywords:** Cardiovascular biology, Kidney, Experimental models of disease, Preclinical research, Physiology

## Abstract

Acute kidney injury (AKI) is common in patients, causes systemic sequelae, and predisposes patients to long-term cardiovascular disease. To date, studies of the effects of AKI on cardiovascular outcomes have only been performed in male mice. We recently demonstrated that male mice developed diastolic dysfunction, hypertension and reduced cardiac ATP levels versus sham 1 year after AKI. The effects of female sex on long-term cardiac outcomes after AKI are unknown. Therefore, we examined the 1-year cardiorenal outcomes following a single episode of bilateral renal ischemia–reperfusion injury in female C57BL/6 mice using a model with similar severity of AKI and performed concomitantly to recently published male cohorts. To match the severity of AKI between male and female mice, females received 34 min of ischemia time compared to 25 min in males. Serial renal function, echocardiograms and blood pressure assessments were performed throughout the 1-year study. Renal histology, and cardiac and plasma metabolomics and mitochondrial function in the heart and kidney were evaluated at 1 year. Measured glomerular filtration rates (GFR) were similar between male and female mice throughout the 1-year study period. One year after AKI, female mice had preserved diastolic function, normal blood pressure, and preserved levels of cardiac ATP. Compared to males, females demonstrated pathway enrichment in arginine metabolism and amino acid related energy production in both the heart and plasma, and glutathione in the plasma. Cardiac mitochondrial respiration in Complex I of the electron transport chain demonstrated improved mitochondrial function in females compared to males, regardless of AKI or sham. This is the first study to examine the long-term cardiac effects of AKI on female mice and indicate that there are important sex-related cardiorenal differences. The role of female sex in cardiovascular outcomes after AKI merits further investigation.

## Introduction

Acute kidney injury is common and is associated with the development of chronic cardiovascular disease^1–6^. There has been a growing appreciation that sex as a biological variable should be taken into account when designing pre-clinical research studies^7,8^, however the renoprotective effect of estrogen on ischemic AKI has complicated the inclusion of both sexes in animal studies^9–13^.

Murine studies evaluating short term cardiac outcomes after AKI have shown that cardiac dysfunction is associated with mitochondrial injury, increased oxidative stress and impairment in energy utilization^14–18^. Clinically, AKI is also increasingly acknowledged as a risk factor for long-term cardiovascular disease, including heart failure and stroke^3,4,19,20^. We have previously demonstrated diastolic dysfunction and perturbations in energy metabolism in the heart early and late after AKI in a murine model of bilateral ischemia–reperfusion injury in male mice^18,21^. In our recently reported 1-year study of ischemia–reperfusion AKI in male mice, we demonstrated persistent diastolic dysfunction after AKI that preceded the development of hypertension. At the end of the 1-year study, male mice had reduced levels of cardiac ATP, which is a potential cause of diastolic dysfunction. The effect of AKI on long-term cardiorenal outcomes in female mice have not been studied, even though it is widely recognized that both males and females should be included in pre-clinical studies.

In this study, we performed a 1-year study utilizing our murine model of bilateral ischemia–reperfusion AKI in females to investigate sex as a biologic variable in the cardiorenal sequelae of AKI. The current study compares the outcomes in female mice at 1 year to our recently published data in male mice 1 year after AKI^21^. At the time of the male study, this female study arm was included to assess the potential effect(s) of sex on major outcomes. Guidelines have recently been established in reporting un-pooled data with regards to sex to improve scientific rigor^22^. We hypothesized that in a matched model of AKI—in which the severity of AKI was matched between the sexes—females would have similar cardiorenal outcomes as males.

## Results

### Survival rates following AKI and Sham

Mortality rates for both sexes were 20–25% following bilateral ischemia–reperfusion injury. In females, 4/19 (21%) of AKI mice died within the initial 48-h post-operative period, and an additional 8/19 (42%) were excluded from the study based on the 24-h BUN and SCr results indicating insufficient degree of AKI was achieved (BUN < 70 mg/dL and SCr < 0.7 mg/dL); 1/7 (14%) of sham mice died, and this death occurred during a 6 month blood pressure reading. In males, 4/13 (23%) died within 48-h of injury, and an additional 1/13 (7%) was excluded for having achieved insufficient degree of AKI; 1/6 (16%) of the sham group did not awake from anesthesia after the sham procedure.

### Matched model of AKI in male and female mice

Table 1 reports the mean measured transcutaneous glomerular filtration rates (tGFR), after AKI in males and females at Baseline, Day^7^, Day^14^, Month^1^, Month^3^, Month^6^, Month^9^ and Month^12^. The serial tGFR data demonstrate that the increased duration of ischemia in females (34 min) compared to males (25 min) resulted in a similar degree of kidney injury with no significant differences in measured tGFR throughout the 1-year study. After having established a matched functional injury model in males and females, we set out to assess the effect of sex on the cardiorenal sequelae.

**Table 1:** Normalized transcutaneous glomerular filtration rate (units = (µL/100 g body weight)/minute) in male and female AKI cohorts throughout the 1 year study.

Time after AKI	Males (mean ± SEM)	Females (mean ± SEM)	P value
Baseline	866.8 ± 44.4	1018 ± 91.7	0.20
Day^7^	308.3 ± 53.6	390.9 ± 199.2	0.57
Day^14^	454.0 ± 56.5	607.6 ± 102.4	0.29
Month^1^	428.1 ± 43.7	506.1 ± 164.0	0.52
Month^3^	500.6 ± 43.2	559.3 ± 68.7	0.55
Month^6^	501.8 ± 32.6	415.1 ± 86.9	0.28
Month^9^	453.3 ± 45.2	496.4 ± 52.1	0.61
Month^12^	476.5 ± 45.6	409.1 ± 75.6	0.43

Demonstration of matched model of AKI between male and female cohorts. In the 1 year study, males underwent 25 min of bilateral ischemia; Females underwent 34 min. Measured transcutaneous glomerular filtration rate (tGFR), normalized for weight, demonstrate a matched model of AKI between the male and female cohorts, with no significant differences in tGFR at any of the time points throughout the 1 year study (n = 7–11).

### Kidney function assessment 1 year after injury in female mice

**Figure 1:**
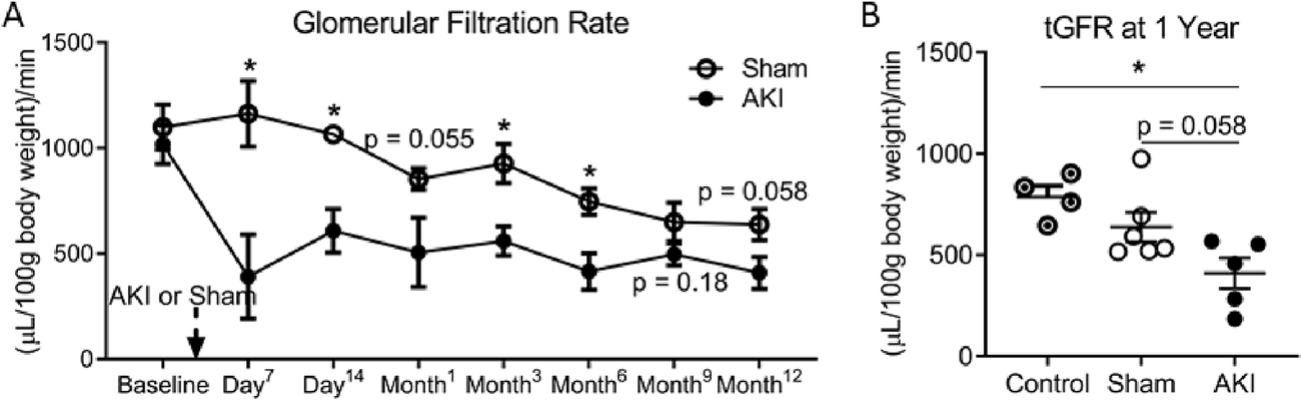
Measured transcutaneous glomerular filtration rate (tGFR) in females after AKI. Female mice underwent 34 min of bilateral renal ischemia or sham. (**A**) AKI resulted in a persistent decrease in tGFR compared to sham throughout the study. (**B**) One year after AKI, tGFR was reduced compared to healthy age-matched controls. (n = 5–7) *Signifies P < 0.05.

**Figure 2:**
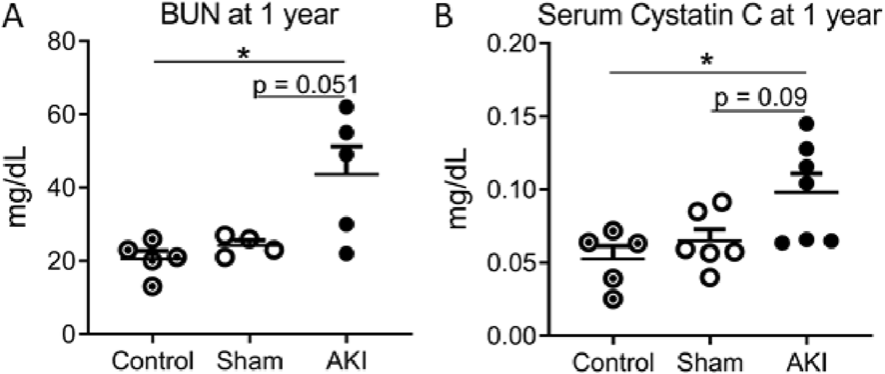
Serum biomarkers of kidney function in females 1 year after AKI or sham, compared to healthy age-matched controls. 12 months after AKI, blood urea nitrogen (**A**) and serum Cystatin C (**B**) were elevated compared to controls, and approached significance compared to shams. (n = 5–7) *Signifies P < 0.05.

In females, the measured kidney function demonstrated a reduction in tGFR in the AKI cohort compared to sham throughout the study duration, with statistical significance reached at the 7- and 14-day, and 3- and 6-month time-points (Fig. 1A). At 1 year, the reduction in tGFR approached statistical significance between AKI and sham cohorts (P = 0.058), with the AKI groups having lower tGFR compared to healthy age-matched controls (Fig. 1B). With regards to serum biomarkers of kidney function, both the BUN (Fig. 2A) and serum cystatin C (Fig. 2B) were increased in the AKI cohort compared to healthy-aged female controls, but not compared to sham (BUN: P = 0.051; Cystatin C: P = 0.09). The serum creatinine measurements at 1 year all fell below the detection limit for HLPC (0.2 mg/dL) (data not shown). These findings match the functional and biomarker assessments in the male arm of the 1-year study^21^.

### Markers of kidney inflammation and fibrosis 1 year AKI in female mice

**Figure 3:**
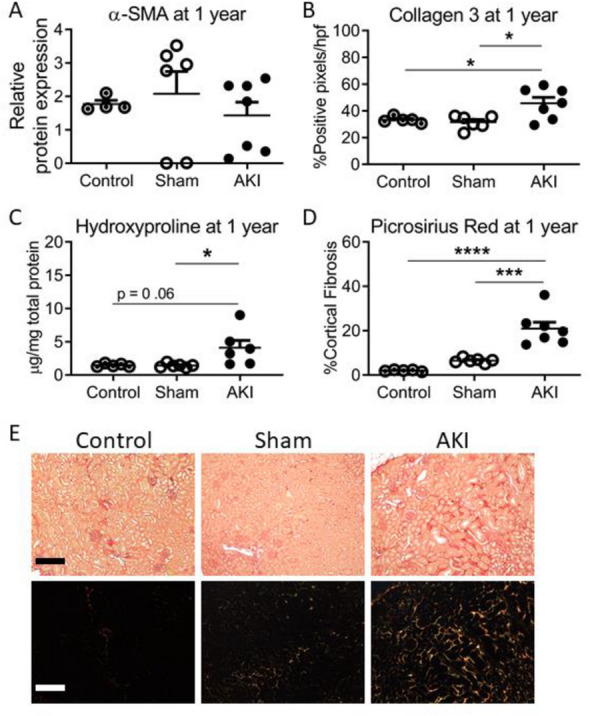
Markers of kidney inflammation and fibrosis in females 1 year after AKI or sham, compared to healthy age-matched controls. Quantification of α-SMA (**A**) and of fibrosis via Collagen 3 immunohistochemistry (**B**), Hydoxyproline content (**C**) and Picrosirius Red (**D**), showed no difference in α-SMA content, but an increase in fibrosis at 12 months in the AKI compared to control and sham cohorts. (n = 5–7) *, **, *** and ****Signify P < 0.05, P < 0.01, P < 0.001 and P < 0.0001, respectively. (**E**) Representative Picrosirius Red images. Top panel, light microscopy; Bottom panel, polarized images. Magnification, ×200. Scale bar = 10 µM.

There was no difference in expression of α-smooth muscle actin (α-SMA) amongst the healthy age-matched controls, sham or AKI cohorts 1 year after AKI (Fig. 3A). Markers of fibrosis were higher in the AKI cohort compared to both the sham and control cohorts assessed by quantification of Collagen 3 immunohistochemistry (Fig. 3B), hydroxyproline content (Fig. 3C) and Picrosirius Red staining, (Fig. 3D), with representative light and polarized images in Fig. 3E. Overall, these data mirror the 1 year α-SMA and fibrosis marker findings in the male cohorts^21^, however, the female AKI cohorts demonstrated a higher absolute quantification of Collagen 3, and Picrosirius Red staining, but not hydroxyproline content, compared to males (Supplementary Table 1).

### Effect of sex on cardiac outcomes after AKI

**Figure 4:**
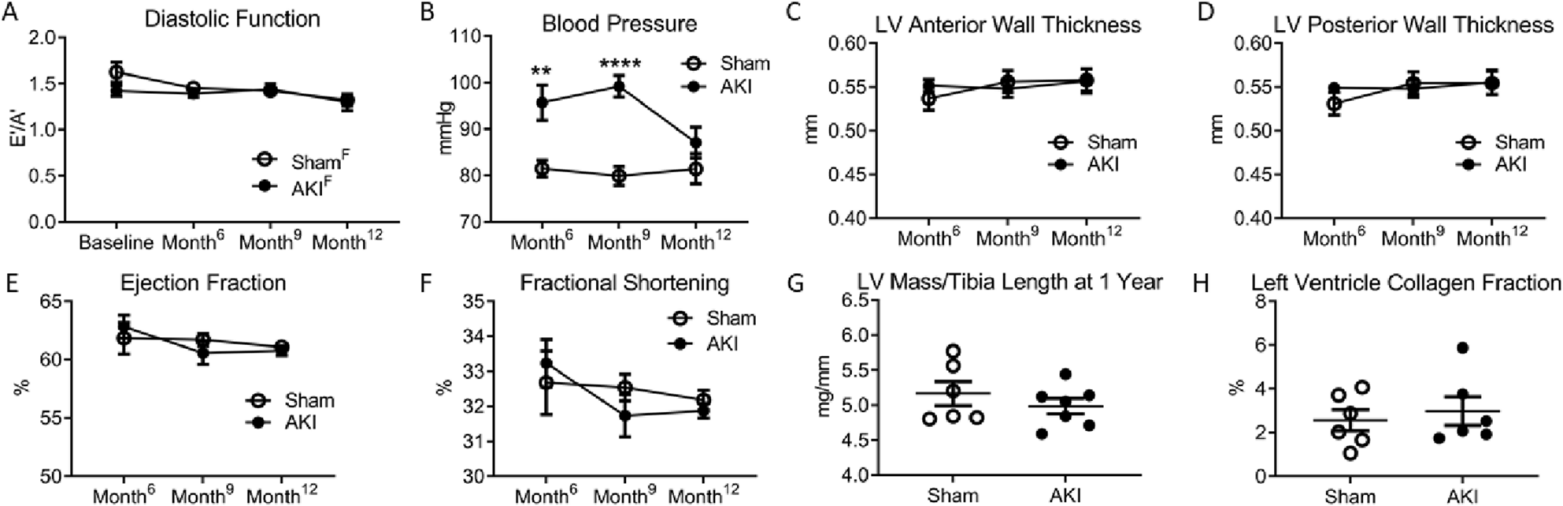
Cardiovascular assessment in females after AKI. (**A**) Females with or without AKI had preserved diastolic function throughout the 1 year study. (**B**) Cohorts who underwent AKI had elevated mean arterial blood pressure measurements compared to sham cohorts 6 and 9 months after procedure, but there was no significant difference in blood pressure between the two cohorts at 1 year. (**C**) There was no difference between AKI and sham cohorts left ventricle (LV) end diastolic anterior (**C**) or posterior (**D**) wall thickness, ejection fraction (**E**) or fractional shortening (**F**) throughout the study. There was no difference in LV mass corrected for tibia length (**G**) or LV collagen fraction (H) 1 year after AKI or sham. (n = 5–7) **P < 0.01; ****P < 0.0001.

Serial echocardiograms were performed to measure mitral annular tissue velocities during early (E′) and late (A′) phases of diastole. A low E′/A′ ratio represents impairment in left ventricular wall relaxation during diastole. Female mice had preservation of cardiac function throughout the 1-year study (Fig. 4A). Mean arterial blood pressure measurements demonstrated hypertension in females after AKI at 6 and 9 months (Fig. 4B) but not at 1 year. There was no difference in end diastolic left ventricular (LV) anterior (Fig. 4C) or posterior (Fig. 4D) wall thickness, ejection fraction (Fig. 4E) or fractional shortening (Fig. 4F) between sham and AKI cohorts at 6-, 9- or 12-months post-procedure. There was no difference in LV mass, controlled for tibia length (Fig. 4G) or LV collagen content (Fig. 4H) at 1 year amongst the AKI or sham cohorts. Overall, females had preserved diastolic function and the hypertension resolved by 1-year, compared to the male AKI counterparts that had diastolic dysfunction which preceded persistent hypertension^21^.

### Effect of sex on the cardiac and plasma metabolome

**Figure 5:**
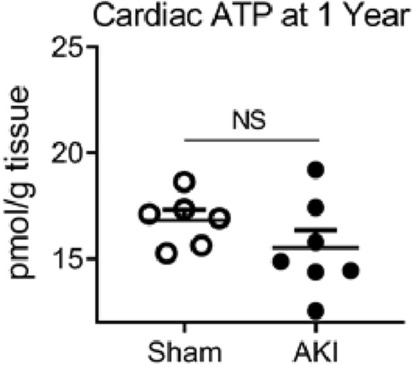
Cardiac ATP levels in females 1 year after AKI or sham procedure. There was no significant difference in Cardiac ATP (n = 6–7).

There were 26 metabolites altered in the heart and 42 in the plasma in females compared to males. Metabolomics pathway enrichment analysis using these metabolites revealed enrichment in females for pathways involving arginine metabolism and amino acid related energy production in both the heart and plasma, and glutathione metabolism in the plasma (Table 2). Unlike males^21^, one year after AKI, female cardiac ATP was preserved compared to sham (Fig. 5). Kidney ATP was also measured on the 1-year cohorts. There was no significant difference in measured ATP in kidney tissue with regards to either AKI or sex 1 year after injury (Supplementary Fig. 2).

**Table 2:** MetaboAnalyst pathway enrichment in plasma and heart tissue.

Pathway	Total	Hits		Raw p	FDR	Impact
**Plasma**						
Arginine and proline metabolism	38	7	2.18E−05		1.54E−03	0.26
Glutathione metabolism	28	6	3.67E−05		1.54E−03	0.30
Purine metabolism	65	8	1.11E−04		3.12E−03	0.09
Arginine biosynthesis	14	4	2.58E−04		5.42E−03	0.14
Alanine, aspartate and glutamate metabolism	28	5	4.41E−04		7.40E−03	0.36
Glycolysis/gluconeogenesis	26	4	3.12E−03		0.044	0.25
**Heart**						
Arginine biosynthesis	14	5	1.30E−06		1.09E−04	0.52
Alanine, aspartate and glutamate metabolism	28	4	8.32E−04		0.035	0.33
Histidine metabolism	16	3	1.81E−03		0.049	0.31
Arginine and proline metabolism	38	4	2.69E−03		0.049	0.20
Nitrogen metabolism	6	2	3.60E−03		0.049	0.00
d-Glutamine and d-glutamate metabolism	6	2	3.60E−03		0.049	0.50
beta-Alanine metabolism	21	3	4.07E−03		0.049	0.11

MetaboAnalyst Pathway Enrichment Analysis in plasma and heart tissue, for analytes significantly different between male versus female mice following acute kidney injury. False Discovery Rate (FDR) significance set at < 0.05.

### Effect of sex on cardiac mitochondrial function

**Figure 6:**
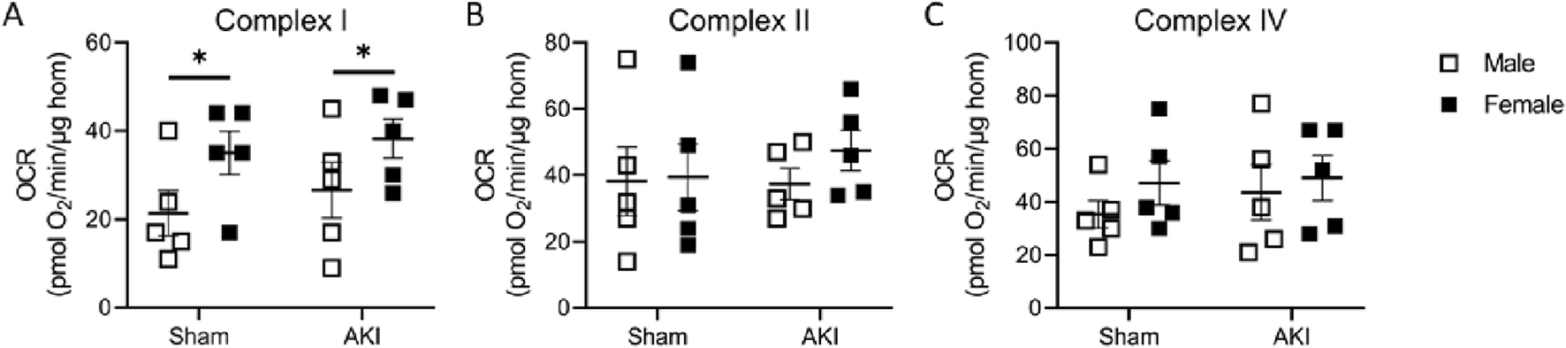
Cardiac mitochondrial respiratory function in males and females 1 year after AKI or sham procedure. (**A**) Females demonstrated higher oxygen consumption rate (OCR) in Complex I compared to males, regardless of AKI or sham status. There was no difference in Complex II (**B**) or Complex IV (**C**) with respect to either sex or AKI. (n = 5–7) *Signifies P < 0.05.

In order to assess whether changes in mitochondrial function explained the sex differences in cardiac ATP levels and diastolic function, we performed respirometry on the 1-year frozen cardiac tissue from all male and female AKI and sham cohorts to assess electron transport chain Complex I, II and IV activity^23^. Two-way ANOVA demonstrated higher Complex I activity in females compared to males (P = 0.028); there was no significant difference between AKI and sham animals. There were no statistical differences noted for either AKI vs sham or male vs female for Complexes II or IV (Fig. 6). Supplementary Fig. 1 shows the respirometry in kidney tissue of the corresponding cohorts. There was no significant difference in the oxygen consumption rate of Complexes I, II or IV with regards to either sex or AKI status in the kidneys, however the OCR of Complex I approached significance (P = 0.06) with females trending higher than males (Supplementary Fig. 2).

### Effect of sex on lipid peroxidation

**Figure 7:**
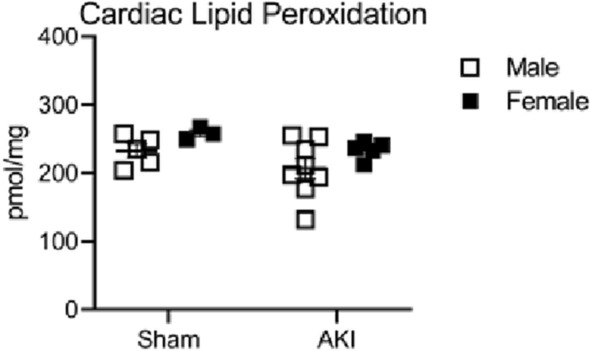
Cardiac lipid peroxidation in males and females 1 year after AKI or sham procedure. In the heart, there was no difference in lipid peroxidation with respect to either sex or AKI. (n = 5–7).

In order to assess whether there were differences in cardiac oxidative damage between males and females 1 year after AKI or sham, we measured MDA to assess lipid peroxidation in the 1-year cardiac samples. Figure 7 demonstrates no significant difference in lipid peroxidation in the heart 1 year after AKI or sham, regardless of sex.

### Systemic inflammation after AKI in females

**Figure 8:**
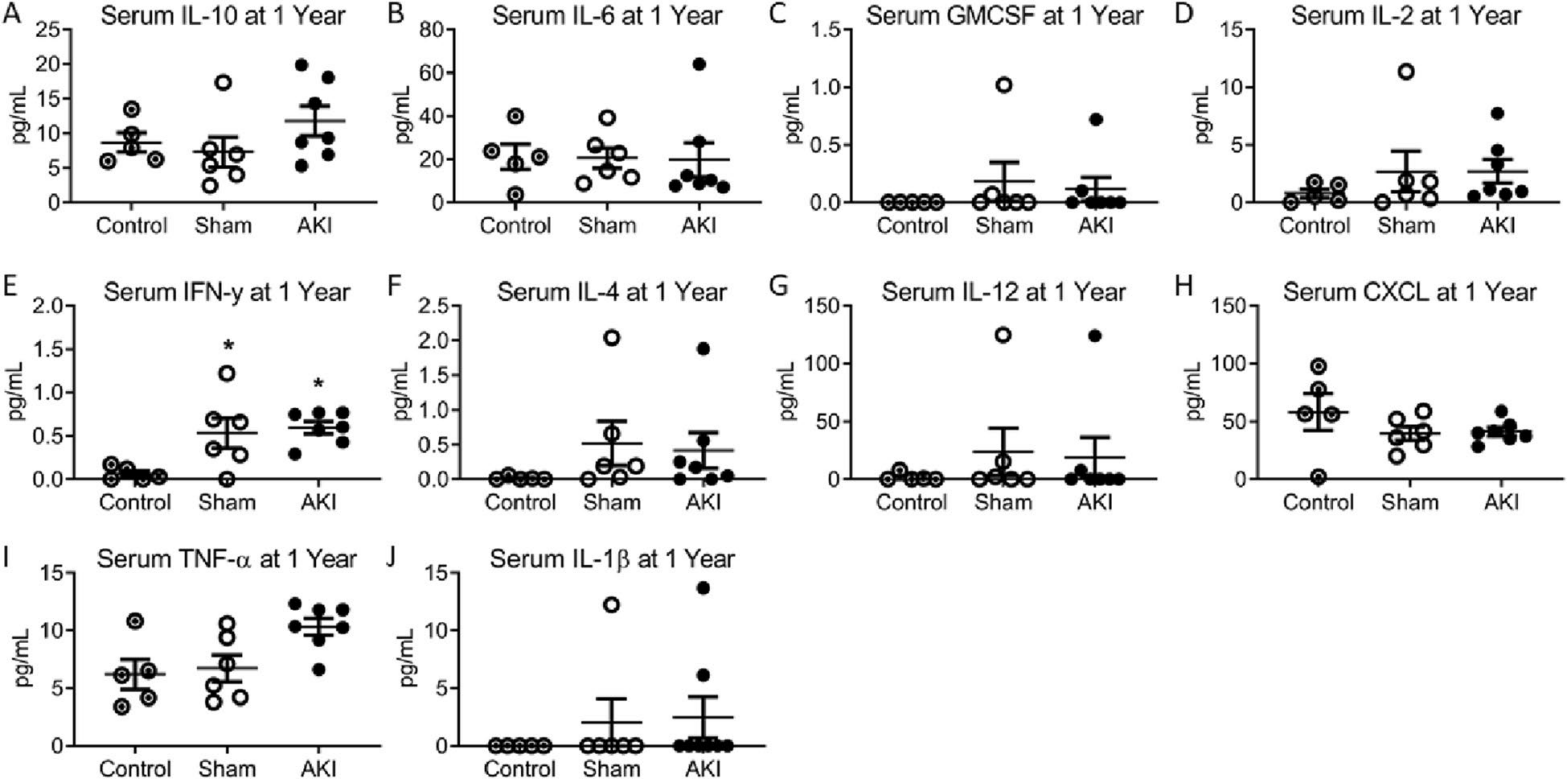
Serum cytokine profile in females 1 year after AKI. (**A**), IL-6 (**B**), GMCSF (**C**), IL-2 (**D**), IFN-γ (**E**), IL-4 (**F**), IL-12 (**G**), CXCL1 (**H**), TNF-α (**I**) and IL-1β (**J**). (n = 5–7) *Signifies P < 0.05 compared to control.

We next sought to determine whether systemic inflammation could be contributing to alterations in cardiovascular outcomes. We have previously demonstrated that AKI results in short, but not long-term inflammation in male mice^21,24,25^. Figure 8 demonstrates an increase in serum IFN-γ in the AKI and sham cohorts compared to 12-month age-matched controls, but no other significant differences were identified in the 10 measured serum cytokines at 1-year. There were no significant differences in the measured cytokines 1 year after AKI between males and females.

### Effect of AKI on weight

Female mice demonstrated a reduction in weight 1 year after AKI compared to sham (Supplementary Fig. 3A), however, there was no significant difference in gastrocnemius muscle mass (Supplementary Fig. 3B). These results match the findings in male cohorts^21^.

## Discussion

In this 1-year study, we investigated the role of female sex in cardiorenal outcomes after AKI. Females required increased ischemia duration compared to males to elicit similar functional injury (assessed by measured tGFR). Key findings of our study are summarized in Table 3 and include:

1. At 1 year, fibrosis markers were increased in females after AKI compared to sham. In the AKI cohorts, quantified values of fibrosis markers were higher in females than males.
2. Females developed hypertension after AKI (similar to males), but unlike males, the hypertension resolved 1 year after injury.
3. Unlike male AKI cohorts, females did not develop diastolic dysfunction, nor reduced cardiac ATP 1 year after AKI.
4. In the heart, 1 year after AKI, females demonstrated pathway enrichment in arginine metabolism and amino acid energy production compared to males. Female hearts also demonstrated higher Complex I mitochondrial function than males, irrespective of AKI or sham procedures.

**Table 3:** Summary of major outcomes after AKI in male and female cohorts.

Outcome measure in AKI cohorts	Males 25 min ischemia	Males^ITF2357^ 25 min ischemia	Females 34 min ischemia
Cardiac ATP		Normal	Normal
Diastolic function		Normal	Normal
Serial blood pressure measurements		Normal	
Blood pressure at 1 year		Normal	Normal
Cardiac Metabolomics Pathway enrichment compared to AKI males	*Control*	Amino Acid energy production Glutathione metabolism	Arginine metabolism Amino Acid energy production
Plasma Metabolomics Pathway enrichment compared to AKI males	*Control*	Amino Acid energy production Pentose phosphate pathway Purine metabolism Glycolysis/gluconeo-genesis	Arginine metabolism Amino Acid energy production Glutathione metabolism
tGFR			
Kidney fibrosis			
Weight			

Summary of major cardiorenal, metabolic and growth outcomes in AKI cohorts including males, males treated with ITF2357 and females. Adapted from Soranno et al. JACC^21^.

In the current study, we set out to investigate the sex dimorphism of cardiorenal outcomes in a matched model of AKI in wild-type adult intact mice. Females required 34 min of ischemia duration, compared to 25 min in males to overcome the kidney-protective effects of female sex in ischemic AKI. With these ischemia times, GFR between males and females undergoing AKI were similar throughout the 1-year study. The GFR data demonstrates that—with regards to kidney function—the model of AKI was matched between the sexes. Notably, females had increased kidney fibrosis markers at 1 year compared to males, indicating that female sex does not protect against renal scarring, which may be an effect of the prolonged ischemia time. We have previously demonstrated the effect of increasing ischemia duration on renal fibrosis^26^. Further investigations are warranted to determine the variability in kidney fibrosis and cardiac outcomes based on sex in a matched ischemia duration model (rather than matched degree of injury) and to study the effects of both sex hormones and sex chromosomes.

In male mice, we have demonstrated a decrease in cardiac ATP levels as early as 4 h after AKI^18^, and 1 year after AKI^21^. In the current report, we demonstrate that female mice maintained normal cardiac function and cardiac ATP levels 1 year after AKI. Decreased ATP in the heart is a recognized cause of diastolic dysfunction^27^. Thus, preserved diastolic function in female mice may be due, at least in part, to the protective effect of female sex on the cardiac metabolome. Here, we also demonstrate that hypertension had resolved by 1-year in females while males demonstrated persistent hypertension after AKI^21^. The role of sex on cardiorenal outcomes after AKI remains under investigated^28^.

Others have previously demonstrated that estrogen protects against oxidative stress and may have an anti-inflammatory effect on the metabolome^29,30^. Our 1-year serum cytokine data indicate that female sex does not mitigate cardiovascular disease via systemic-anti-inflammatory effects. Our metabolomics results suggest that female sex protects the cardiac metabolome following AKI. The effect sex has on the cardiac metabolome after AKI has not been previously reported. Using metabolomics, we found that in the plasma, glutathione metabolism was enriched (which could relate to reactive oxygen species), and in both plasma and heart tissue, arginine and nitrogen metabolism were enriched (which could relate to reactive nitrogen species). Both suggest that mitochondria-related oxidative stress is differentially affected between the sexes. Alterations in oxidative stress are a recognized contributor to the development of kidney disease^31,32^. l-arginine substrate is required for estrogen to elicit protective nitric oxide, and its depletion has been suggested as a mechanism by which post-menopausal women undergo accelerated cardiovascular disease^33^. Other enriched pathways included those related to energy metabolism via glycolysis, gluconeogenesis, and purine metabolism, as well as metabolism of the amino acids alanine, aspartate, and glutamate which feed directly into the mitochondrial tricarboxylic acid (TCA) cycle.

Taken together, the metabolomic data and cardiac ATP levels suggest that the degree of mitochondrial dysfunction may play a role in differences between male and female mice with AKI. Mitochondria generate reactive oxygen species (ROS), and ischemia–reperfusion AKI is known to increase ROS and lead to mitochondrial injury^34–36^. While male and female C57BL/6J mice have equivalent amount of mitochondria, sex differences in number and quality have been described in both murine and clinical studies, with females having more cardiac mitochondria and superior morphology compared to males^37–39^.

Estrogen has previously been shown to reduce oxidative stress in murine models of chronic disease, and AKI has been shown to cause increased ROS months after injury^29,40^. Estrogen has been shown to mitigate oxidative stress, and Singh et al. have demonstrated that estradiol infusion protects against renal injury by reducing oxidative stress following ischemia–reperfusion injury in rats^41,42^. Either the presence of estrogen, or the absence of testosterone could be protective factors affecting cardiovascular outcomes, and sex chromosome complement may also play a role independent of gonadal hormones. Emerging data suggest that the number of X chromosomes may have a deleterious effect on cardiovascular outcomes following direct cardiac injury^43–45^. Future investigations that unconfound the effects of sex hormones and sex chromosomes could reveal mechanistic insights to the protective effect of female sex on cardiac function but not kidney fibrosis.

While the AKI cohorts appeared healthy after their initial recovery, their growth parameters were affected long-term, with AKI mice weighing significantly less than healthy and sham controls 1 year after injury. These data suggest that clinical studies are warranted to determine the effects of AKI on growth parameters, particularly in children. Clinically, AKI has been shown to increase fracture risk in adult and pediatric patients^46–48^. Further investigations are needed to determine if the musculoskeletal changes reported here are related to changes in adiposity or lean bone and muscle mass.

This is the first long-term study evaluating sex as a biological variable in a matched model of AKI in female and male mice. The strengths of our study include the measurement of kidney function rather than reliance upon serum biomarkers and histological outcomes to achieve a matched model of AKI. Our study also has several limitations. First, the cohort sizes are small, and the risks of Type I and Type II errors exist. Second, we did not measure sex hormone levels throughout the study. Third, the healthy age-matched 1-year controls were aged at Jackson laboratories and shipped to the University of Colorado 2 weeks prior to sacrifice. These controls therefore spent most of their lives in a different vivarium and at a different altitude, which may affect some of our findings. Further studies investigating the impact of renal development and aging, such as in pups or aged mice may improve the translatability of the findings. Finally, AKI is a heterogeneous disease with numerous causes; while ischemia–reperfusion injury is highly clinically relevant, this injury model represents only one cause of AKI.

In summary, we have demonstrated important differences in long-term cardiorenal outcomes between male and female mice after a matched degree of AKI. Future studies are needed to investigate the mechanism by which female sex protects against diastolic dysfunction after AKI but not hypertension or kidney fibrosis. Such investigations are warranted as they may identify protective sex biases and provide novel therapeutic targets to improve cardiorenal outcomes for both sexes.

## Methods

### Animal model of acute kidney injury

Surgical procedures were performed on 8-week-old mice female C57BL/6J mice (Jackson Laboratories, Bar Harbor, ME.): (1) sham operation and (2) ischemic AKI. Mice were randomly dichotomized to undergo either sham or AKI procedure. For all procedures, mice were anesthetized with a Ketamine (VetOne, MWI, Boise ID) Xylazine (VetOne, Bimed-MTC Animal Health Inc., Cambridge, ON) cocktail. Mice were shaved and cleaned, then placed on a heating pad maintained at 36–40 °C for the entire procedure. A ventral midline incision was made, and the renal pedicles were identified. In the ischemic AKI group, both renal pedicles were clamped for 34 min for female mice. Male mice, reported elsewhere^21^, underwent 25 min of ischemia duration. After clamp removal, kidneys were observed for restoration of blood flow by the return to original color. Sham surgery consisted of the same procedure except clamps were not applied. The abdomen was closed in two layers, first muscle layer using 4-0 Vicryl (Ethicon, Sommerville, NJ), then skin layer using 4-0 Sofsilk (Covidien, Mansfield, MA). After closure, mice were administered 750 µL warmed saline/Buprenorphine Hydrocholoride (0.3 mg/mL, PAR Pharmaceuticals, Chestnut Ridge NY) at a 1:10 dilution subcutaneously then placed in a sterilized cage on a heating pad until awake. Once awake mice were then given moistened chow, sterile nesting material and placed on a rack, half on a heating pad set at low for two days. After two days, mice were moved to normal housing racks. 750 µL of warmed normal saline were administered subcutaneously to each mouse for 5 days post-operatively. Animals that did not exhibit sufficient AKI based on the post-operative day 1 BUN and SCr were excluded from the study. Criteria for study inclusion in the AKI groups were BUN > 70 mg/dL or SCr > 0.7 mg/dL. Thereafter, investigators were blinded to the sample group allocation during analysis. Mice were maintained on a standard diet, and water was freely available. Aged 12-month study controls were aged at Jackson Laboratories and transported to the University of Colorado 2 weeks prior to sacrifice.

### Measurement of glomerular filtration rate

Transcutaneous glomerular filtration rate (tGFR) measurements were performed using a transdermal continuous renal function monitor (MediBeacon GMBH, Manheim, Germany). Mice were weighed and anesthetized then shaved dorsally, in the thoracic region of the back until all fur was removed. Devices were placed on the shaved region using patches supplied by MediBeacon and Surgical tape (Micropore, 3 M Heath Care, St Paul, MN). A mixture of normal saline and FITC was injected intravenously via tail vein. Devices were left on for at least 65 min while mice were placed in individual cages. Measures were taken to ensure minimal stimulus including lower lighting and minimal sound. tGFR measurements were taken at Baseline (3–7 days prior to surgery), then serially until sacrifice. Mice were allowed at least 3 days of recovery after tGFR readings before surgeries were performed.

### Collection and preparation of tissue samples

Serum was collected at 24 h post-AKI/sham procedure and at sacrifice, the kidneys, liver, spleen, partial lung, heart, and muscle were collected and processed as previously described^21^.

### Assessment of kidney function and kidney injury

BUN and SCr were assayed using a BioAssay Systems QuantiChrom™ Urea Assay Kit Cat: (DIUR-500) and Pointe Scientific Creatinine (Enzymatic) Reagent Kit (Cat: C7548-480) for the 24-h inclusion time point. Due to extensive hemolysis in the male 12-month groups, 12-month SCr in both sexes was measured using the Element DC Veterinary Chemistry Analyzer (Serial #:73110474, Manufacturer: Fujifilm, Stamford, CT, supported by Heska, Des Moines, IA) via the Comparative Pathology Core at UC Denver. Cytokines were measured by a customized U-Plex Biomarker-1 (Cat#: K15069L-S, Meso Scale Discovery, Rockville, MD) following the manufacturer's instructions as previously reported^21^.

### Western Blot

Kidneys were homogenized and analyzed as previously reported with alpha-smooth muscle actin (α-SMA) primary antibody (ab32575, Abcam, Cambridge, MA, 1:5000)^26^. Blots were normalized using the Revert Total Protein Stain (Cat #: 926-11010, Licor, Lincoln, NE) as a loading control and performed following manufacturer’s instructions.

### Histology

Kidney tissue was processed for Picrosirius Red and Collagen 3 immunohistochemistry as previously reported^21^. The percentage of collagen tissue in the left ventricle was quantified on the 1-year cardiac tissue samples as previously described^49^.

### Hydroxyproline assay

Hydoxyproline content was measured in the left kidney from each sample as previously reported^26^.

### Echocardiography and blood pressure measurement

Serial transthoracic echocardiography and Doppler analyses were performed using Vevo2100 instruments (VisualSonics, Toronto, Canada), and mean arterial blood pressure measurements using a non-invasive computerized tail-cuff system (CODA High Throughput System, Kent Scientific Corp, Torrington, CT) were performed as previously described^49^.

### Determination of myocardial and kidney ATP content

Pre-weighed left ventricle (LV) and kidney tissue was processed using commercially available reagents as per manufacturer’s instructions (Abcam; ab833355). Briefly, LV or kidney tissue samples in ATP assay buffer were homogenized using Dounce homogenizer. The supernatants obtained after clarification was subjected to deproteinization prior to the assay. Sample reactions were incubated for 30 min at room temperature, followed by fluorescence intensity measurements on a microplate reader (Ex/Em = 535/587 nm). ATP concentrations were normalized to tissue weight.

### Metabolomics

Frozen heart and plasma samples were processed as previously reported^18^. Mean values for AKI^M^, AKI^F^, Sham^M^, and Sham^F^ (M = male; F = female) were calculated, and Student’s t-test was used to compare each group to the others as previously reported^21^. The two main outcomes assessed were (1) Metabolites that were shared between the AKI^F^ versus AKI^M^ analysis AND Sham^F^ versus Sham^M^ analysis, to determine metabolite enrichment shared in both analyses as related to sex (F = female; M = male). (2) Metabolites that were uniquely significant to either AKI^F^ versus AKI^M^ analysis OR Sham^F^ versus Sham^M^ analysis to determine significant differences in response to sex that are not shared between the two groups. These experiments were conducted in both heart tissue and plasma samples.

### Mitochondrial respiration

Respirometry in frozen samples was performed as previously described on frozen ventricular and kidney samples from both the female and male cohorts that underwent sham or AKI procedure^23^. Briefly, the oxygen consumption rate was measured for Complexes I, II, and IV, normalizing samples based on protein content and mitochondrial DNA.

### Lipid peroxidation

Lipid peroxidation was measured on the 1-year heart samples from male and female cohorts following manufacturer’s instructions (Lipid Peroxidation (MDA) Assay Kit, Sigma-Aldrich, St. Louis MO).

### Statistics

Cohorts contained n = 5–11. Sham cohorts had fewer (n = 5) than the AKI cohorts to account for anticipated increased mortality rates post-operatively in the AKI groups. Comparison amongst three or more groups was performed via ANOVA with Tukey post-hoc analysis. Comparison between two groups was performed via unpaired t-tests assuming Gaussian distribution with Welch’s correction. 2-way ANOVA was performed on mitochondrial respiration data with respect to both AKI and sex. Two-sided Grubbs’ test was utilized to determine outliers (Alpha = 0.05). Data in figures represent mean ± SEM.

### Study approval

All studies were approved by the University of Colorado’s Animal Care and Use Committee and adhered to the National Institutes of Health Guide for the Care and Use of Laboratory Animals and the ARRIVE guidelines.

## Supplementary Information

Supplementary Information.

## Data Availability

The data that support the findings of this study are available from the corresponding author (DES), upon reasonable request.
