# Supplementary material for: Female and male mice have differential longterm cardiorenal outcomes following a matched degree of ischemia–reperfusion acute kidney injury
Source: Sci Rep. 2022 Jan 12;12:643. doi: 10.1038/s41598-021-04701-x (PMC8755805; doi:10.1038/s41598-021-04701-x)
Supplement: Supplementary file 1 — Supplementary Information. [file 41598_2021_4701_MOESM1_ESM.docx]

**Supplemental Material Table of Contents:**

**Table 1:** Quantification of kidney fibrosis markers in male and female cohorts 1 year after AKI.

**Figure 1:** Kidney mitochondrial respiratory function in males and females 1 year after AKI or sham.

**Figure 2**: Kidney ATP levels in males and females 1 year after AKI or sham

**Figure 3:** Growth outcomes in females after AKI

**Supplemental Table 1: Quantification of kidney fibrosis markers in male and female cohorts 1 year after AKI.**

| **Fibrosis Marker** | **Males (Mean ± SEM)** | **Females (Mean ± SEM)** | **P value** |
| --- | --- | --- | --- |
| **Collagen 3**  (%positive pixels/hpf) | 32.9 ± 2.9 | 46.6 ± 4.7 | **0.013** |
| **Hydroxyproline**  (µg/mg total protein) | 2.3 ± 0.26 | 4.1 ± 1.12 | 0.173 |
| **Picrosirius Red**  (%Cortical fibrosis) | 11.9 ± 2.3 | 20.9 ± 2.9 | **0.027** |

Supplementary Table 1. Markers of kidney fibrosis were higher 1 year after injury in females that underwent 34 minutes of ischemia, compared to males with 25 minutes of ischemia. Quantification of both Collagen 3 Immunohistochemistry and Picrosirius Red staining were significantly increased in females compared to males. There was no significant difference in hydroxyproline content. (n = 7-11) HPF = high-powered field.

**Supplemental Figure 1: Kidney ATP in males and females 1 year after AKI or sham.**

**
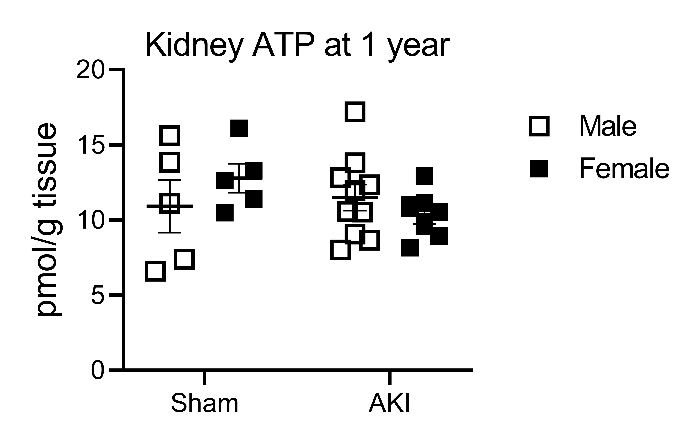
**

Supplemental Figure 1. Kidney ATP measurements in males and females 1 year after AKI or sham procedure. There was no statistically significant difference in measured ATP with regards to sex or AKI status. (n = 5-7).

**Supplemental Figure 2: Kidney mitochondrial respiratory function in males and females 1 year after AKI or sham.**

**
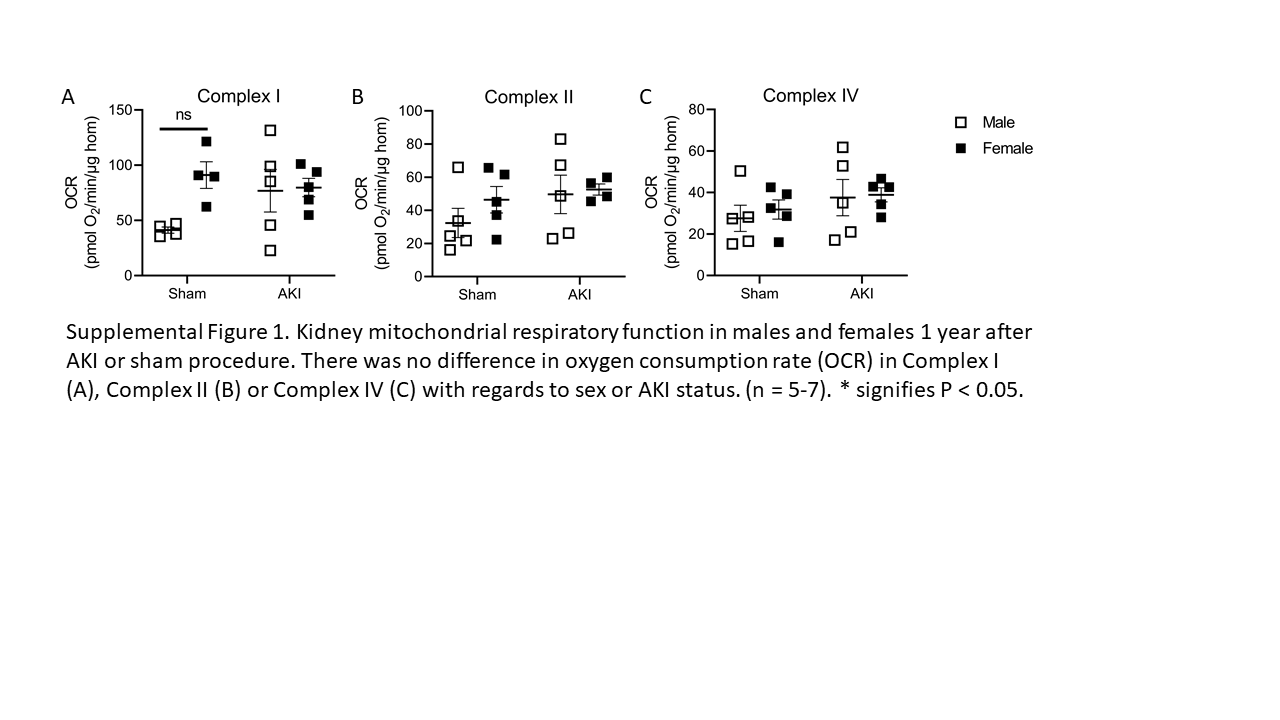
**

Supplemental Figure 2. Kidney mitochondrial respiratory function in males and females 1 year after AKI or sham procedure. There was no difference in oxygen consumption rate (OCR) in Complex I (A), Complex II (B) or Complex IV (C) with regards to sex or AKI status. (n = 5-7).

**Supplemental Figure 3:** Growth outcomes in females after AKI.
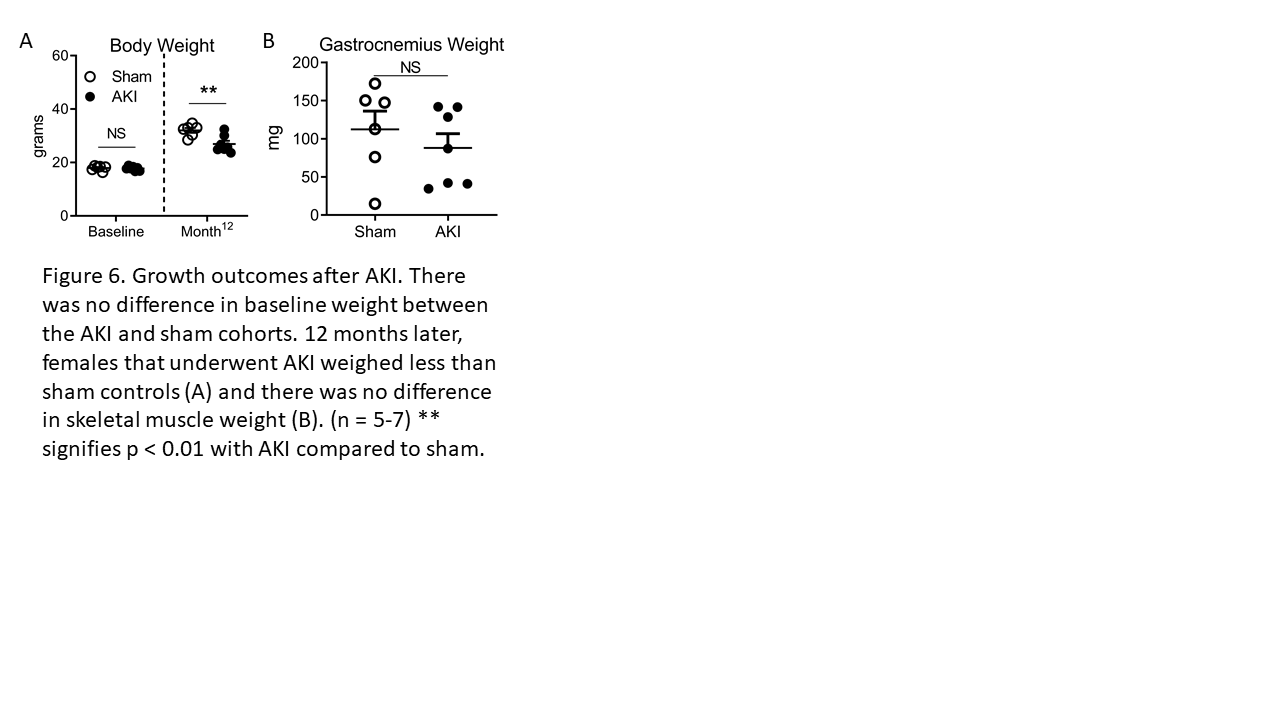


Supplemental Figure 3. Growth outcomes in females after AKI or sham. There was no difference in baseline weight between the AKI and sham cohorts. 12 months later, females that underwent AKI weighed less than sham controls (A) and there was no difference in skeletal muscle weight (B). (n = 5-7) ** signifies p < 0.01 with AKI compared to sham.
